# Application of metagenomic next-generation sequencing in the diagnosis of post-stroke infections: a case series study using multiple sample types

**DOI:** 10.3389/fcimb.2024.1386377

**Published:** 2025-01-08

**Authors:** Xiaopu Chen, Yong Liang, Wei Yang, Wenzhen He, Zhiqiang Xing, Shunxian Li, Shaoyu Cai, Jiping Fu, Xiaotang Peng, Manli Chen, Jiaming Wu

**Affiliations:** ^1^ Department of Neurology, The First Affiliated Hospital of Shantou University Medical College, Shantou, Guangdong, China; ^2^ Department of Research and Development, Shenzhen Xbiome Biotech Co. Ltd., Shenzhen, China; ^3^ GeneMind Biosciences Company Limited, Shenzhen, Guangdong, China; ^4^ Department of Diagnosis Technology Transformation, SK Medical Technology Co, Ltd, Beijing, China

**Keywords:** next-generation sequencing, stroke-related infections, diagnostic effect, treatment, department of neurology

## Abstract

**Background:**

Metagenomic high-throughput sequencing (mNGS) represents a powerful tool for detecting nucleic acids from various pathogens, such as bacteria, fungi, viruses and parasites, in clinical samples. Despite its extensively employed in the pathogen diagnosis for various infectious diseases, its application in diagnosing stroke-related infection, and its potential impact on clinical decision-making, anti-infection treatment, clinical intervention, and patient prognosis remain insufficiently explored. Additionally, while mNGS offers promising potential, it facts limitations related to sensitivity, specificity, cost, and standardization, which could influence its integration into routine clinical practice.

**Methods:**

We retrospectively analyzed 18 stroke patients admitted to the First Affiliated Hospital of Medical College of Shantou University from January to February 2023, comparing culture-based methods with mNGS detection, and assessing its significance in etiological diagnosis. Additionally, we evaluated the performance differences among various sequencing platforms.

**Results:**

Among the 18 stroke patients enrolled, pulmonary infections were identified in 7 cases, urinary tract infections in 1 case, central nervous system infections in 10 cases, and combined pulmonary and central nervous system infections in 2 cases, with 2 cases yielding negative results. mNGS detected pathogens in 13 cases, aligning with clinical diagnoses (75% concordance), whereas culture-based methods yielded positive results in only 6 cases (22% concordance). Importantly, for 9 of the 18 patients, adjustments to anti-infective treatment regimens based on mNGS results led to improved symptomatic relief and infection control. This suggests that mNGS can contribute to more timely and precise treatment modifications, particularly for infections with low pathogen loads, potentially enhancing clinical outcomes.

**Conclusion:**

Our findings highlights the utility of mNGS in diagnosing stroke-associated infections by providing a more comprehensive etiological diagnosis compared to traditional method. While mNGS shows promise in enhancing diagnostic accuracy and guiding clinical treatment, it high cost and technical challenges need addressing before widespread clinical adoption. Future research should focus on optimizing mNGS protocols, integration it with convertional diagnostic tools, and evaluating its cost-effectiveness and clinical impact through larger, multicentric studies.

## Introduction

Cerebrovascular disease, a prevalent clinical condition predominantly instigated by cerebral ischemia or hemorrhage, manifests with sudden onset and rapid progression, posing a serious threat to patient safety if left untreated (Wang, 2018; QiaoZhen et al., 2019). Current clinical strategies for stroke management primarily revolve around enhancing cerebral blood circulation, thrombolytic therapy, and antiplatelet interventions. Despite these efforts, complications such as ventilator-associated pneumonia, acute upper gastrointestinal bleeding and catheter-associated bloodstream infection often ensue. Among these complications, Stroke-associated infection (SAI) emerges within 7 days post-stroke, with pulmonary infection and urinary tract infection being the most prevalent (Hannawi et al., 2013; Liu et al., 2018; Teh et al., 2018).

The incidence of stroke-associated pneumonia ranges from 7% to 38%, with gram-negative bacteria such as *Klebsiella pneumoniae* and *Acinetobacter baumannii* being the most commonly isolated pathogens (Ji et al., 2013b; Smith et al., 2015; Yu et al., 2016; Teh et al., 2018). Additionally, studies have indicated a prevalence of gram-positive bacteria, including Staphylococcus aureus and Staphylococcus epidermidis, along with fungi like Candida albicans in stroke-related infections (Ji et al., 2013a, 2014; Jin et al., 2017; Liying and Cortez, 2021; Haijin, 2023). Notably, the emergence of gram-negative bacteria as predominant pathogens underscores the importance of targeted antimicrobial selection based on sensitivity profiles (Liying and Cortez, 2021; Haijin, 2023). Meanwhile, the advent of SAI complicates the clinical management, exacerbating the condition and substantially elevating mortality and morbidity risks.

Recognizing the pivotal role of early and accurate pathogen identification in guiding targeted anti-infective therapy, innovative diagnostic approaches are imperative. Metagenomic high-throughput sequencing (mNGS) technology, capable of unbiased detection of bacteria, fungi, viruses, and other pathogens in clinical samples, has garnered substantial recognition for its diagnostic utility in infectious diseases (Jin et al., 2017). Notably, conventional diagnostic methods, including microbial culture, antigen-antibody assays, and nucleic acid detection, have been augmented by mNGS in recent years (Wilson et al., 2014). However, while mNGS has demonstrated efficacy in diagnosing nervous system infections (Fan et al., 2019; Wilson et al., 2019; Xing et al., 2020; Zhang et al., 2020; Lei et al., 2021), its application in SAI diagnosis warrants further exploration.

This study aims to assess the diagnostic performance of mNGS in SAI detection, comparing it with conventional methods and evaluating different high-throughput sequencing platforms. Through retrospective analysis of stroke patients admitted to the First Affiliated Hospital of Medical College of Shantou University, we elucidate the role of mNGS in enhancing etiological diagnosis and optimizing clinical treatment regimens. Our findings provide valuable insights into the potential of mNGS to revolutionize SAI management, ultimately improving patient outcomes.

## Methods

### Study design

Patients with a clinical dagnosis of suspected stroke-associated infection (SAI) were randomly selected from the First Affiliated Hospital of the Medical College of Shantou University between January 2023 and February 2023. Eligible participants were aged between 18 and 80 years.

Exclusion criteria were applied to ensure the integrity of the study. Patients who voluntarily withdrew from the study were excluded, as were cases with incomplete data for any reason. Additionally, subjects were excluded if detection of pathogens was not possible due to errors in sample collection, preservation, or procedural mistakes, and if re-sampling was not feasible. Participants with inadequate nucleic acid quality or failed experiments that could not be re-sampled were also excluded. Finally, any other individuals deemed inappropriate for continued participation by the researchers were excluded from the study.

Infection-related samples, including blood, cerebrospinal fluid, sputum, and urine samples were collected for culture and mNGS detection. This study adhered to the Declaration of Helsinki and received approved from the Ethics Review Board of the First Affiliated Hospital of Shantou University. Informed consent was obtained from all participating patients, and the samples were used exclusively for this study.

### Sample collection

Patient information, including demographics and laboratory results, was collected from the hospital information system (HIS). Patients were categorized into group based on clinical diagnosis: pulmonary infection, urinary tract infection, and central nervous system infection.

#### Clinical Definitions

A) Pulmonary Infection: Includes:

Post-Stroke Pulmonary Infection: Specifically defined as pneumonia occurring within 7 days following a stroke in non-mechanically ventilated patients.

Standards: Diagnosed based on radiographic evidence of lung infiltrates or consolidation.

B) Urinary Tract Infection (UTI): Encompasses infections involving:

Upper Urinary Tract Infections: Such as pyelonephritis.

Lower Urinary Tract Infections: Such as cystitis.

Standards: Diagnosed based on clinical symptoms (e.g., frequency, urgency, dysuria, or flank pain) in conjunction with urinalysis findings (e.g., pyuria, positive nitrite test) and urine microscopy (e.g., presence of white blood cell casts, bacteria).

C) Central Nervous System (CNS) Infection: Includes infections where pathogens—such as viruses, bacteria, fungi, or parasites—invade the brain, meninges, or spinal cord.

Standards: Diagnosed based on PCR assays. Additional evidence includes elevated white blood cell counts, altered glucose levels, or increased protein levels in CSF.

#### Disease Severity

A) The majority of recruited patients presented with moderate to severe infections, as indicated by clinical evaluations and laboratory results.

B) The definition of urinary tract infections encompasses both upper and lower urinary tract infections.

C) The infections selected for study included:

Pulmonary Infections: post-stroke pneumonia.

Urinary Tract Infections: Infections involving the entire urinary system.

Central Nervous System Infections: Infections affecting the brain, meninges, or spinal cord caused by various pathogens.

### Culture method

Specimens were collected prior to or following initiation of antimicrobial treatment, with the initial specimen ideally obtained at disease onset to minimize contamination. None of the patients had received antimicrobial treatment before specimen collection, ensuring that the samples were not influenced by prior antibiotic use. The culture-based methods employed for pathogen identification adhered to standard clinical protocols. These included techniques such as blood cultures for systemic infections, sputum cultures for pulmonary infections, and urine cultures for urinary tract infections. For central nervous system infections, cerebrospinal fluid cultures were used. Each culture type followed established procedures to accurately isolate and identify the suspected microorganisms.

#### Collection of cerebrospinal fluid

The patients were positioned in the decubitus or lateral decubitus position with knee flexion. The collection site was determined at the posterior superior iliac spine and the posterior midline intersection of the spine, typically targeting the 3rd to 4th lumbar spinous process space. Following local anesthesia with 1-2% lidocaine, a sterile needle was vertically inserted into the intervertebral spinous space. Approximately 3 mL of cerebrospinal fluid was aspirated and transferred into a sterile container for culture.

#### Collection of urine specimens

Urine samples were obtained from various sources, including catheters, indwelling catheters and midstream urine. For molecular diagnosis, urine specimens were preferably collected from the first urine voided in the morning. Samples were promptly transported to sterile containers for testing.

#### Collection of lower respiratory tract samples

Under local anesthesia with 2% lidocaine, a bronchoscope was introduced through the nose or mouth. The bronchoscope was directed to severe areas identified through imaging or bronchoscope findings. Bronchial brush, bronchial aspirates and other relevant samples were collected into sterile containers and promptly sent for testing. It is essential to note that bronchoscopic specimens are susceptible to contamination by upper respiratory tract flora, necessitating rigorous quality assessment.

#### Collection of sputum

Patients were instructed on the distinction between sputum and saliva, with denture removal recommended if applicable. After gargling with water to minimize contamination, patients were guided to cough up deep sputum vigorously. Saliva and post-nasal secretions were not considered suitable for sputum testing.

#### Collection of blood samples

Blood culture samples were collected in accordance with antimicrobial drug use labels. It is recommended to obtain 2-3 sets of samples from different sites testing, ensuring comprehensive assessment.

### mNGS detection method

To mitigate the influence of a single sequencing platform, both the BGI MGI 500 and Genemind GenoLab M high-throughput sequencers were employed for mNGS detection.

For the BGI MGI 500 platform, DNA was extracted from tissue samples using the TIANamp Micro DNA kit (DP316, Tiangen Biotech, Beijing, China) according to the manufacturer’s recommendations, fragmented with by Bioruptor (Thermofisher ScSAIntific, Waltham, Massachusetts, USA-RRB-instrument) yielding 200 bp fragments, and subjected to terminal repair, a-tail treatment, and barcode ligation, followed by PCR amplification. Quality control included assessment of fragment size using the Agilent 2100 system and quantification of DNA library concentration using the Qubit dsDNA HS Assay Kit. Libraries were pooled, and single-stranded DNA loops were formed and sequenced using the MGI 500 platform via SE50 mode. High-quality sequencing data underwent preprocessing to remove low-quality reads, adapter contamination, and short reads (< 50 bp). Human sequences were eliminated by aligning to the GRCH38 human reference genome using Burrows-Wheeler Alignment (BWA) (v0.7.13). Subsequently, non-human sequences were aligned to the NCBI NT database using SNAP (v2.0.2). Advanced data analysis included taxonomic annotation, genome coverage/depth calculation, and abundance calculation using internal scripts.

For the Genemind GenoLab M platform, DNA extraction was performed using the Shengkang Medical Universal Pathogen Nucleic Acid Extraction Kit. The extracted DNA was fragmented, and library construction was conducted using the Shengkang Medical Library Kit. Total DNA fragments, ranging from 200-300bp in size, were extracted and processed for library construction using the Shengkang Medical Library Kit. DNA end repair, linker ligation, and PCR amplification were conducted according to kit protocols. Quality control of library and insert fragment sizes was performed using the Agilent 2100 Bioanalyzer. Sequencing of the constructed library was carried out in SE150 mode using the Genemind GenoLab M sequencer, generating a minimum of 20M sequence data. Low-quality, low-complexity, and <70bp sequences were filtered out, and human reference genome sequences were removed. High-quality sequencing data were then compared with the microbial genome database for microbial identification.

The entire process adhered to stringent quality control measures. Clinicians integrated patient history, clinical features, and relevant auxiliary examination results to assess the pathogenicity of mNGS results.

### Statistical analysis

Normal distribution and homogeneity of variance were assessed using the T-test. Differences in continuous variables between groups were analyzed using the Student’s t-test and the chi-square test. Propensity score matching (PSM) was employed to assess the diagnostic effect of NGS accurately. Statistical analysis was conducted using SPSS 26.0(IBM, Armonk, NY) software, with significance set at Pvalue < 0.05.

For bioinformatics analysis, data quality control was performed using Fastp to filter and trim sequencing reads. The BWA (Burrows-Wheeler Aligner) algorithm was employed for sequence alignment, followed by taxonomic classification using Kraken2 to identify microbial species present in the samples. Antimicrobial resistance (AMR) analysis was conducted using tools such as CARD (Comprehensive Antibiotic Resistance Database) to assess the presence of resistance genes in the metagenomic data.

## Results

### Patient and sample characteristics

**Figure 1:**
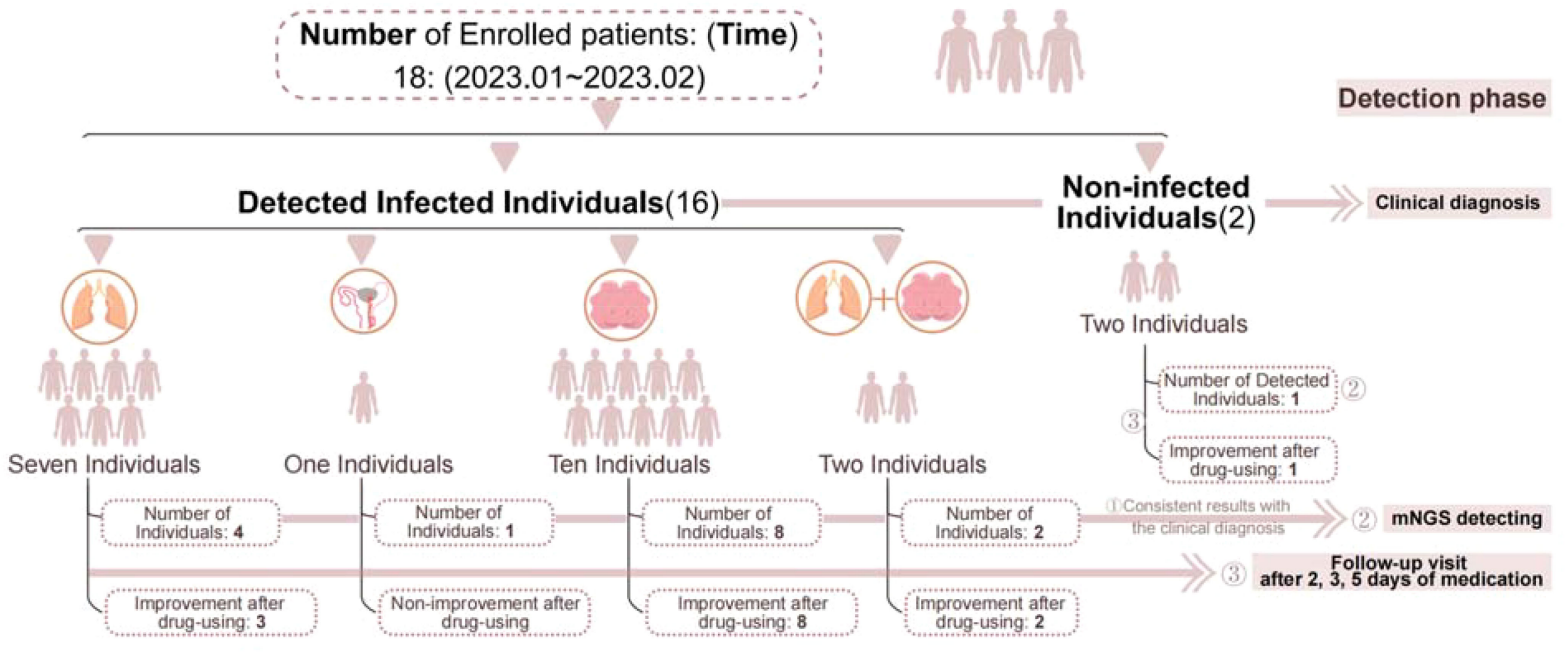
Flow chart of patients included. ① Indicates that both methods detected the infection or confirmed no infection, resulting in consistent outcomes. ② Represents the number of positive samples detected by mNGS. ③ Indicates follow-up monitoring after medication.

The study included a total of 18 patients, comprising 11 men and 7 women, with a mean age of 54. Demographics and pertinent characteristics of the enrolled patients are summarized in **Table 1**. Among them, 7 cases were diagnosed with pulmonary infection, 1 case with urinary tract infection, 10 cases with central nervous system infection, 2 cases with combined pulmonary and central nervous system infections, and 2 cases yielded negative results (**Figure 1**). There were no significant differences observed in sex, age or medical history among the groups.

**Table 1:** Baseline characteristics and clinical indices of patients included.

	Total	Pulmonary	Urinary tract	Central nervous system	Concurrent	Negitibe
Gender (male/female)	11/7	4/3	1/0	5/5	1/1	2/0
Age (Min~Max)	54 (26-85)	53.7 (32-85)	72	47.7 (26-62)	38.5 (32-45)	65 (58-72)
Cardiovascular disease	13/5	5/2	1/0	9/1	2/0	0/2
Cerebrovascular disease	13/5	4/3	0/1	2/8	0/2	2/0
COVID-19	17/1	6/1	1/0	10/0	2/0	2/0

Cardiovascular and cerebral diseases encompass a wide range of conditions that significantly impact public health. Notably, stroke, a major type of cerebrovascular disease, is linked to high morbidity and mortality rates (Johnson et al., 2016; O'Donnell et al., 2016). Furthermore, cardiovascular diseases, including heart attacks and heart failure, remain leading causes of death globally (Murray et al., 2019). The prevalence of these diseases necessitates effective diagnostic and treatment strategies.

Of the 18 patients (**Table 1**), mNGS analysis was conducted on 27 samples, which included various sample types such as sputum, urine, or cerebrospinal fluid (CSF) from the same patient, yielding 19 positive results. One sample was deemed substandard due to sputum contaminated, and 16 samples were deemed clinically significant. The mNGS results were consistent with clinical diagnosis in 14 cases, effectively identifying the actual infection status of the patients. Discrepancies were noted in 4 cases. Among the discordant cases, two were positive for mNGS but considered sample contamination in conjunction with other clinical indications (**Figure 1**).

### Comparison between mNGS detection and culture-method

**Figure 2:**
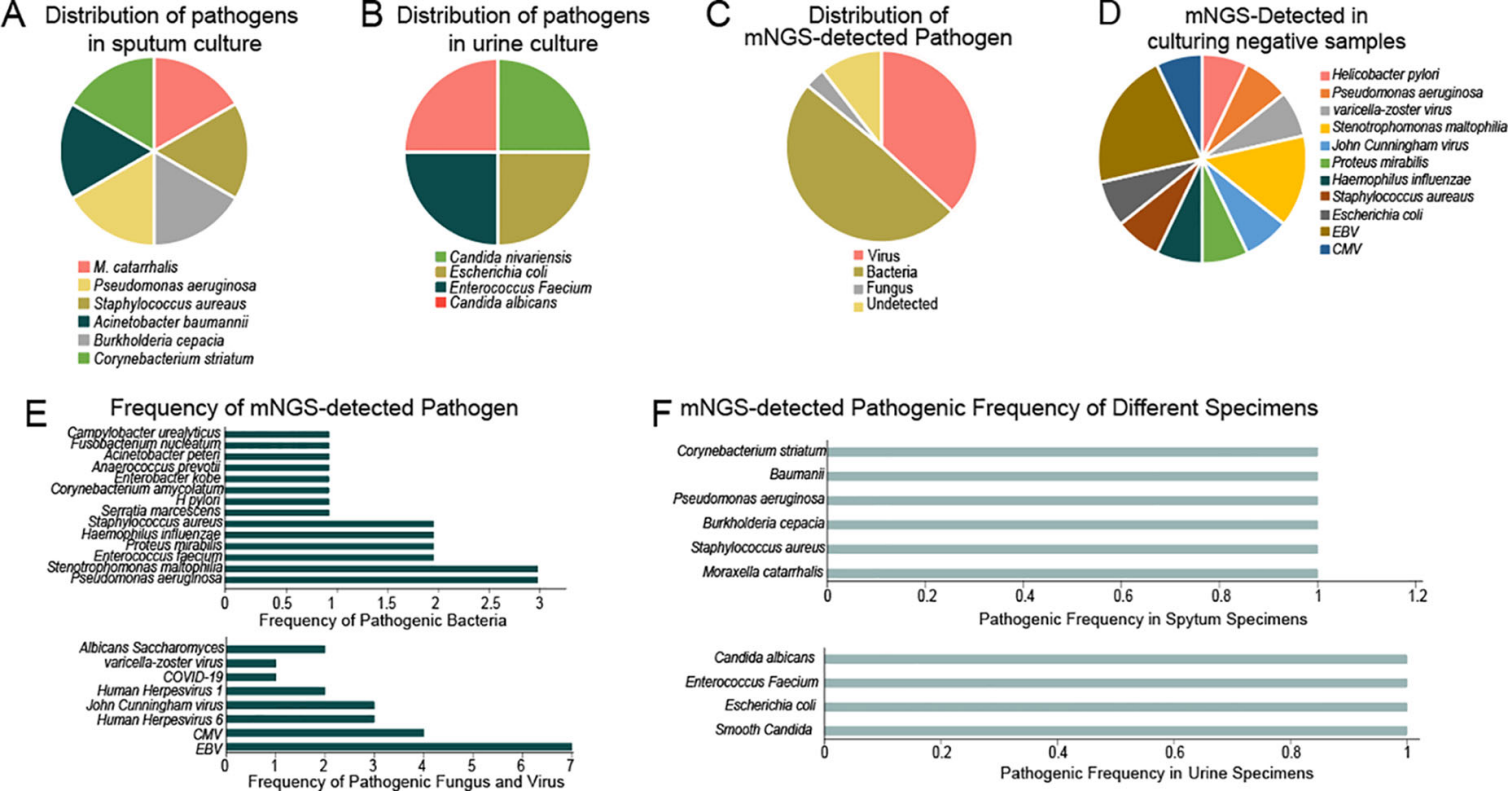
Distribution of pathogens in different specimens by different detecting methods. **(A)** Distribution of pathogenes in sputum culture; **(B)** Distribution of pathegenes in urine culture; **(C)** Distribution of mNGS-detected Pathogenes; **(D)** mNGS detected in culturing negative samples; **(E)** Frequency of mNGS-detected pathogen; **(F)** mNGS detected pathogenic frequency of different specimens.

In this study, simultaneous cerebrospinal fluid (CSF) and blood samples yielded negative results, while sputum and urine samples showed varying detection rates in both testing methods (**Figure 2**). In cases where the test results return negative for a specific sample, the combination of clinical characteristics allows for the exclusion of infection in the sampled area of the infected patient. Therefore, this negative result aligns with the clinical presentation (**Figure 2D**). However, contamination during clinical collection was a concern for sputum and urine samples, necessitating further clinical correlation for interpreting positive results.

Among the 18 patients, 14 showed negative results in culture, of which 6 were also negative in mNGS. Excluding one case considered contaminated, 7 patients tested positive in mNGS, resulting in a positive rate of 53% and a diagnostic agreement of 73%.

Viral infections were detected in 10 out of 18 cases, with a detection rate of 55% and a concordance rate of 90%. Hence, mNGS exhibited superior performance in detecting low-load pathogens, particularly in virus diagnosis, providing more sensitive results.

These findings suggest that while mNGS demonstrates superior performance in detecting low-load pathogens, particularly viruses, its efficacy in identifying bacterial pathogens is hindered by the bacterial load. mNGS can effectively detect low-load viruses because viral genomes often have unique sequences that can be amplified even in small quantities, allowing for reliable identification. Additionally, viruses typically replicate within host cells, resulting in higher concentrations of viral nucleic acids in samples.

In contrast, low-load bacterial pathogens present challenges due to their diverse genetic makeup and the potential for contamination. Bacterial DNA may be present in lower concentrations, and if the pathogen load is too low, there may not be enough genetic material to achieve reliable detection. Some bacteria may also exist in a viable but non-culturable state, complicating detection by mNGS. This highlights the need for improved methods to optimize mNGS for low-load bacterial infections, as addressing these challenges will be essential for enhancing its diagnostic capabilities in clinical settings.

### Enhanced pathogen detection through metagenomic high-throughput sequencing

Our study utilized both culture-based methods and BGI SE50 sequencing to identify pathogens in sputum and urine samples, firstly. The pathogens identified by traditional culture-based methods were all cultivable types. In our results, culture-based methods identified six bacterial species (including M. catarrhalis, Staphylococcus aureaus, Burkholderia cepacian, Pseudomonas aeruginosa, Acinetobacter baumannii, and Corynebacterium striatum) in sputum (**Figure 2A**) and four bacterial species (including Candida nivariensis, Escherichia coli, Enterococcus Faecium, and Candida albicans) in urine (**Figure 2B**). In contrast, BGI SE50 sequencing detected a broader range of microorganisms, including viruses, bacteria, fungi, and others (**Figure 2C**). And our results fully demonstrate this point. BGI SE50 sequencing detected the presence of the following 13 pathogens in negative control samples, encompassing both cultivable bacterial pathogens and non-cultivable viruses (such as varicella-zoster virus, John Cunningham virus, EBV, CMV). These pathogen detection results based on negative control samples can serve as indicators for further infection in specific anatomical sites of infected patients, thus holding clinical significance (**Figures 2D, E**). Furthermore, our comparison of corresponding samples, where traditional methods and mNGS were both employed, demonstrated that mNGS could detect numerous low-level pathogenic bacteria and viruses, highlighting its superior sensitivity compared to culture-based methods (**Figure 2F**).

### Influenced clinical intervention protocols by mNGS outcomes

In 9 of the 18 patients, adjustments to the treatment regimen based on the mNGS test results resulted in improved symptomatic relief of the infections, especially for patients with low-load pathogen infections, earlier and more accurate adjustments can be made (**Figure 1**). It highlights the effectiveness of mNGS in guiding treatment decisions for better clinical outcomes.

### Performance comparison

#### Performance in data quality

**Figure 3:**
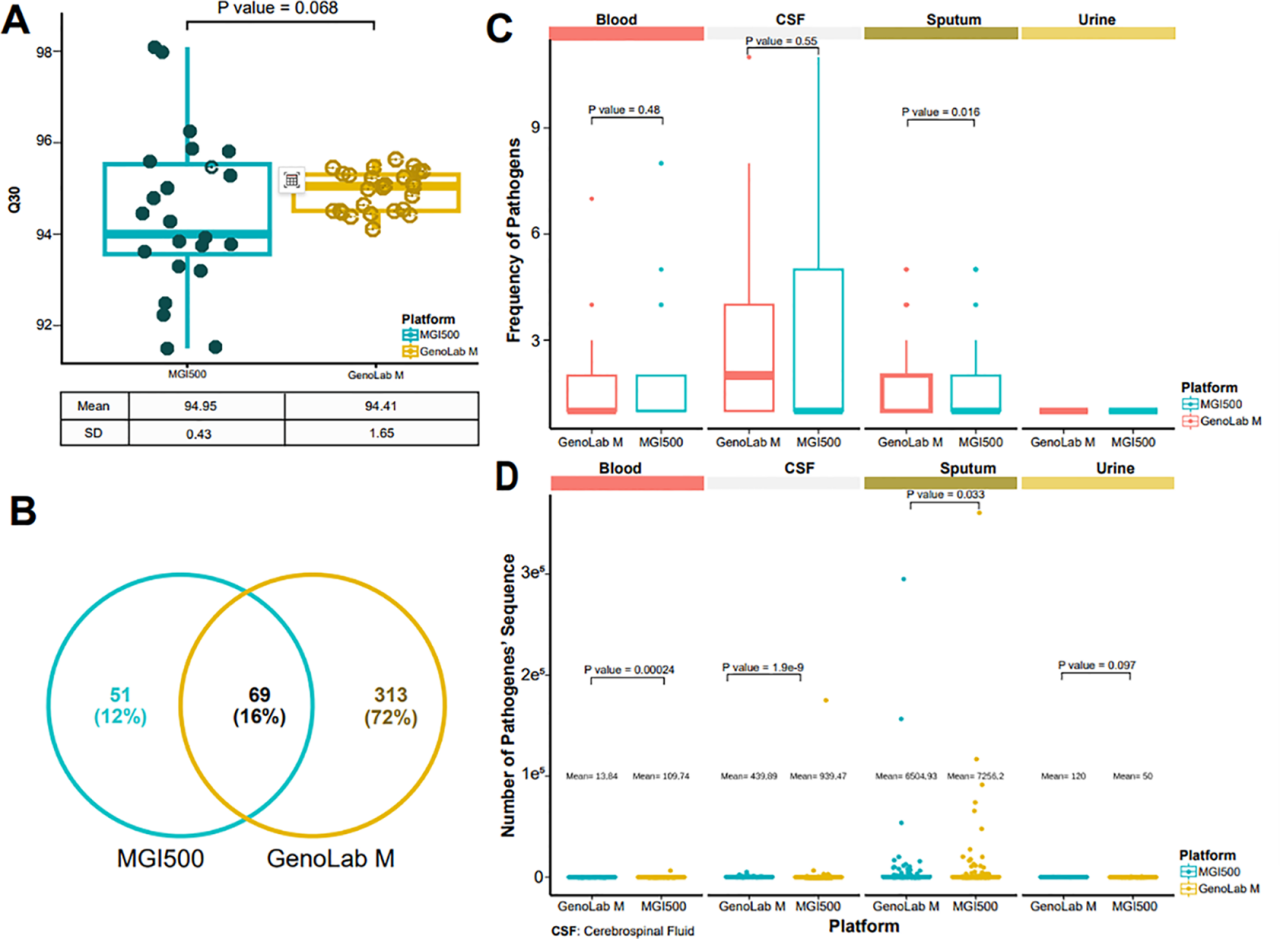
MGI 500 and GenoLab-M detected data quality. **(A)** Distribution of reads with a quality score of 30 across two sequencing platforms; **(B)** Detected pathogens based on metagenomic next-generation sequencing (mNGS) from the two platforms; **(C)** Number of pathogenic bacteria identified from four sample types using the data output from both platforms; **(D)** Distribution of sequence counts for pathogenic bacteria derived from the data output of the two platforms across the four sample types.

Taking into account the positive correlation between sequence length and matching accuracy, we incorporated the GenoLab M sequencer and employed the long-read mode SE150 for mNGS. Our rationale was to assess whether longer sequence lengths could augment the accuracy and sensitivity of pathogen detection. Both platforms demonstrated comparable levels of sequencing data quality across all sequencing data, as indicated by similar Q30 scores. However, the overall data quality of the GenoLab M platform exhibited slight superiority and robustness compared to that of the MGI200 platform (**Figure 3A**). For all the detected pathogens by the MGI200 and GenoLab M sequencers, a total of 69 were identified by both platforms. Notably, the GenoLab M platform revealed a greater diversity of unique pathogens, totaling 313, surpassing the number identified by the MGI200 platform (**Figure 3B**). Despite sequencing four different clinical infection samples, both platforms demonstrated comparable levels of detected fragments, as evidenced by, similar frequency number of detected pathogens (**Figure 3C**) and similar data volume levels of detected pathogens (**Figure 3D**). However, the detected pathogenes of the GenoLab M platform was slightly superior to that of the MGI200 platform.

#### Performance in microbiological testing

**Figure 4:**
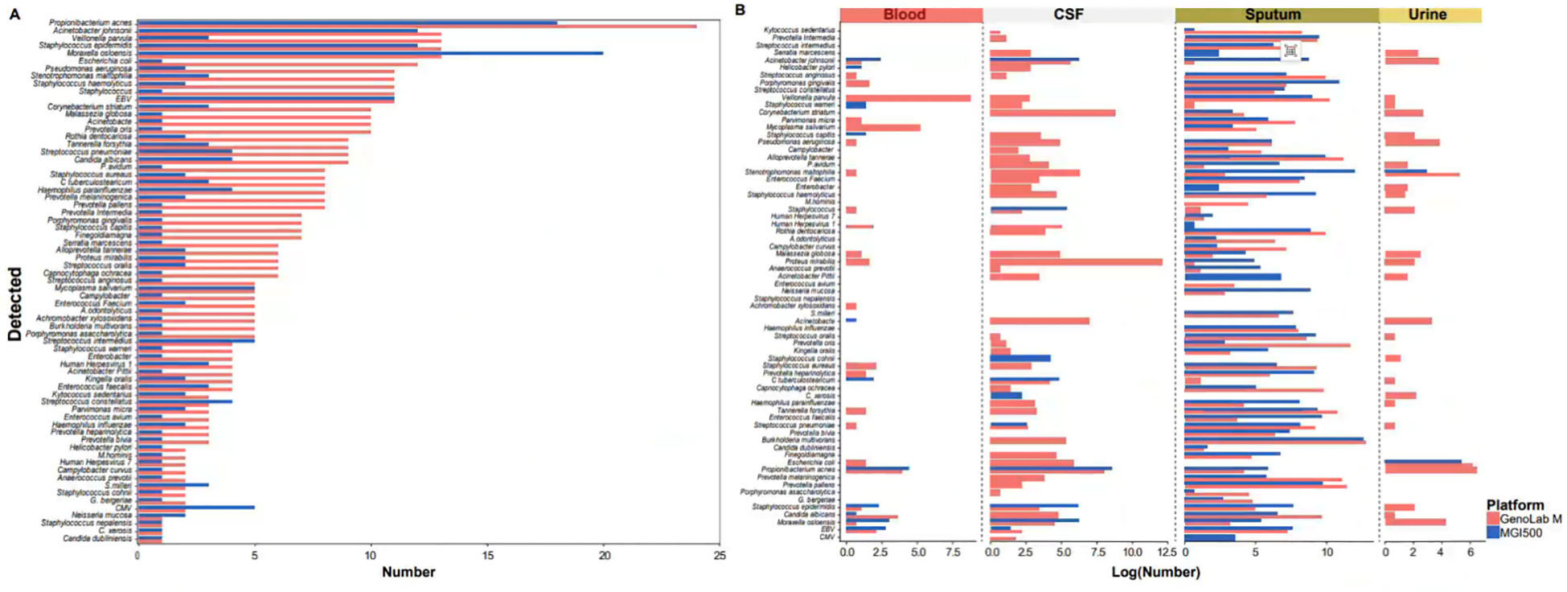
MGI 500 and GenoLab-M detected the difference of microorganisms. **(A)** Types and number of pathogenic microorganisms detected in all samples based on sequencing data from the MGI500 and GenoLab-M platforms; **(B)** Types and number of pathogenic microorganisms detected in the four sample types based on sequencing data from the MGI500 and GenoLab-M platforms.

Subsequently, our analysis assessed significant disparities in the performance of the two platforms in detecting clinically relevant bacteria, fungi, and viral species. Compared to the MGI200 platform, the GenoLab M platform’s long-read sequencing mode demonstrated higher sensitivity and detected a greater number of pathogenic microorganisms. Notably, among the pathogens detected by both platforms, the GenoLab M platform exhibited a higher frequency of pathogen detection compared to the MGI200 platform (**Figure 4A**). Interestingly, in various infection samples, the microbial species detected in sputum samples showed minimal differences between the MGI200 and GenoLab M platforms, with GenoLab M exhibiting slightly superior performance. For instance, GenoLab M detected Staphylococcus aureus and Human Herpesvirus 7, which were not detected by the MGI200 platform. Furthermore, compared to the MGI200 platform, the GenoLab M platform’s sequencing identified a broader range of infection-associated pathogens in blood, urine, and cerebrospinal fluid (CSF) samples, showcasing superior performance (**Figure 4B**).

#### Functional gene testing performance

In addition to species identification, antimicrobial resistance (AMR) gene detection was pursued. Three cases(14,26, and 40) were positive for four AMR genes. Four structural antibiotic resistance genes were identified in the respective species according to CARD (**Table 2**). Notably, the mecA gene abundance was 372.3701359, indicating MRSA’s (Methicillin-Resistant Staphylococcus aureus) resistance to all beta-lactam antibiotic. While this case underscores the potential of mNGS in detecting AMR genes, challenges remain. including detecting First, limitations in sequence read depth from the host hindered the detection of allelic variations in AMR genes. Second, short-read sequencing data lack the context necessary to accurately determine the genomic positions of resistance genes. Additionally, distinguishing between AMR genes carried by plasmids and those carried by chromosomes remains problematic. Furthermore, even if an AMR gene is detected, its contribution to disease resistance may be hindered by factors such as poor expression, silencing, or inactivation. Future studies will prioritize the analysis of AMR genes using NGS technology.

**Table 2:** List of AMR genes.

Cases	Genes of Antibioticresistance	The number of sequenceschecked out	Possible source species
14	*AAC (6”)*	30	*Enterococcus faecium*
26	*SMEDEF, SMEABC*	29,41	*Stenotrophomonas maltophilia*
40	*SMEABC*	8	*Stenotrophomonas maltophilia*

## Discussion

Stroke-associated infections (SAIs) present significant challenges in clinical management due to their potential to exacerbate patient morbidity and mortality. In the current diagnostic and treatment landscape, the prophylactic administration of antimicrobial agents, coupled with the unique characteristics of central nervous system infections, frequently contributes to false-negative outcomes in pathogen detection. While conventional diagnostic methods may yield false-negative results, metagenomic high-throughput sequencing (mNGS) offers a promising alternative for comprehensive pathogen detection. By employing unbiased detection of nucleic acids from various pathogens, including bacteria, fungi, viruses, and parasites, mNGS provides a holistic approach to identifying infectious agents in clinical samples (Zhang et al., 2020; Chen et al., 2022b). This comprehensive assessment addresses the limitations of conventional methods, particularly in cases where microbial cultures may fail to detect low-load or fastidious pathogens due to prior antimicrobial exposure or inherent difficulties in cultivation (Guo et al., 2019; Erdem et al., 2021; Gao et al., 2021; Ge et al., 2021; Guo et al., 2021; Chen et al., 2022a; Gan et al., 2022; Gu et al., 2022). Therefore, the integration of mNGS into diagnostic protocols holds significant promise for improving the accuracy and timeliness of pathogen identification in stroke-associated infections. Our study aimed to evaluate the diagnostic performance of mNGS in SAIs, comparing it with culture-based methods and assessing different high-throughput sequencing platforms.

Our findings reveal the substantial diagnostic utility of mNGS in identifying pathogens across diverse clinical samples, including cerebrospinal fluid, blood, sputum, and urine, obtained from SAI patients. Notably, mNGS exhibited a superior detection rate compared to culture-based methods, particularly in identifying low-load and difficult-to-culture pathogens. This enhanced sensitivity of mNGS is crucial for accurate pathogen identification, guiding targeted antimicrobial therapy and improving patient outcomes. In addition, our study compared the performance of two mainstream high-throughput sequencing platforms, BGI MGI 500 and Genemind GenoLab M, in mNGS analysis. The GenoLab M platform demonstrated slightly superior performance in mNGS, which could be attributed to its longer sequencing reads, leading to increased alignment accuracy and enhanced ability to precisely assign sequences to specific pathogens. Additionally, the higher accuracy of sequencing data may also contribute to this improved performance. It is important to note that this does not imply inferior sequencing data quality from the MGI 500 platform. Rather, our findings suggest that while extending read length in mNGS can enhance diagnostic accuracy, maintaining sequencing data quality is equally essential. Our study results provide evidence in support of this notion to a certain extent.

Furthermore, our study highlights the clinical implications of mNGS in optimizing treatment regimens for SAI patients. By identifying pathogens with high sensitivity and specificity, mNGS enables clinicians to tailor antimicrobial therapy promptly and effectively, reducing the risk of treatment failure and recurrence.

The comparison of two mainstream high-throughput sequencing platforms, BGI MGI 500 and Genemind GenoLab M, underscores the importance of selecting appropriate sequencing technologies for optimal diagnostic performance. While both platforms demonstrated efficacy in pathogen detection, variations in sequencing depth and data quality may influence diagnostic accuracy. Further studies should aim to standardize sequencing protocols and optimize bioinformatics analysis to enhance the reliability and reproducibility of mNGS results.

Despite the promising results, the limitations of our study must be acknowledged. The relatively small sample size introduces a degree of uncertainty into our findings, and as such, our results should be interpreted as a preliminary evaluation of mNGS’s potential in this context. This limitation emphasizes the need for larger-scale studies to further optimize and validate the diagnostic capabilities of mNGS for stroke-associated infections. Future research should consider multicenter designs to enhance sample diversity and strengthen the reliability of the findings.

Additionally, the cost of mNGS remains a significant barrier to its widespread clinical adoption, particularly in resource-limited settings. While the price of mNGS has historically been high, the rapid development of sequencing technologies and the entry of multiple companies into the market may drive costs down. As with other technologies, such as the cost-per-gigabase for sequencing, we anticipate that as mNGS becomes more established, prices will become more aligned with clinical needs. The increasing availability of sequencing instruments and associated products is encouraging, suggesting that mNGS may eventually become a more accessible diagnostic tool.

Moreover, our study also emphasizes the significance of mNGS in detecting antimicrobial resistance (AMR) genes, providing valuable insights into guiding antibiotic therapy and preventing SAI recurrence. Despite the challenges in interpreting AMR gene data, particularly regarding allelic variations and gene expression, mNGS holds promise as a valuable tool for personalized antimicrobial therapy.

It is crucial to discuss the inherent limitations and advantages of mNGS in pathogen detection. While it excels at identifying a diverse array of pathogens, its effectiveness can be hindered by low microbial load, especially for bacterial pathogens. The sensitivity of mNGS for low-load pathogens can vary, posing challenges for detecting bacteria present in minimal quantities. In contrast, mNGS demonstrates exceptional sensitivity for viral pathogens, often outperforming traditional methods in this regard. This duality underscores the importance of ongoing research into optimizing mNGS for low-load bacterial detection.

In summary, while our study emphasizes the substantial potential of mNGS in diagnosing and managing SAIs, it also serves as a call to action for further research and development. By addressing the limitations identified, including sample size, cost, and optimization for low-load pathogen detection, we can pave the way for more effective clinical applications of mNGS. The future holds promise for mNGS as a transformative tool in clinical microbiology, particularly for complex and challenging infections such as SAIs.

## Conclusion

In conclusion, our study underscores the superior diagnostic performance of metagenomic high-throughput sequencing (mNGS) compared to culture-based methods in identifying pathogens associated with stroke-associated infections (SAI). mNGS demonstrates higher sensitivity and shorter turnaround time, particularly beneficial for detecting microorganisms that cannot be cultured, are difficult to cultivate, or are at risk of antibiotic exposure. The comprehensive nature of mNGS allows for unbiased detection of a wide range of pathogens, including bacteria, fungi, viruses, and parasites, overcoming the limitations of traditional culture methods, especially in culture-negative SAI patients. Furthermore, mNGS’s ability to detect antimicrobial resistance genes enhances its utility as a valuable adjunct to microbial culture, aiding in guiding targeted antimicrobial therapy and improving patient outcomes. Overall, our findings highlight the significant potential of mNGS in revolutionizing the diagnosis and management of SAI, paving the way for enhanced clinical decision-making and patient care in this challenging clinical context.

Moreover, the differential performance of various high-throughput sequencing platforms and sequencing mode underscores the importance of selecting appropriate technologies to optimize diagnostic accuracy. Despite the challenges and limitations, mNGS holds immense potential for revolutionizing the diagnosis and management of SAIs, ultimately improving patient outcomes and reducing healthcare burden. Further research efforts aimed at standardization, cost reduction, and validation in larger cohorts are warranted to facilitate the widespread clinical adoption of mNGS in SAI diagnosis and management.

## Data Availability

The data that support the findings of this study have been deposited into the CNGB Sequence Archive (CNSA) of China National GeneBank DataBase (CNGBdb) with accession number CNP0006612. This includes the sequencing reads of all replicates in fastq format.
